# PI16 attenuates response to sorafenib and represents a predictive biomarker in hepatocellular carcinoma

**DOI:** 10.1002/cam4.3331

**Published:** 2020-08-10

**Authors:** Pusen Wang, Zhongyi Jiang, Xueni Liu, Kanru Yu, Chunguang Wang, Hao Li, Lin Zhong

**Affiliations:** ^1^ Department of General Surgery Shanghai General Hospital Shanghai Jiao Tong University School of Medicine Shanghai China

**Keywords:** apoptosis, p38 MAPK, peptidase inhibitor 16, prognosis, sorafenib

## Abstract

Sorafenib has become the only FDA‐approved first‐line therapy for advanced hepatocellular carcinoma (HCC) for more than 10 years, but there is still no validated predictive or prognostic marker. Peptidase inhibitor 16 (PI16) is a functionally unknown gene in cancer research. This study aimed to determine the exact function of PI16 in HCC and whether it can represent as a biomarker for sorafenib response. We found that PI16 was over expressed in HCC tissues vs paired normal tissues. PI16 knockdown sensitize HCC cells to sorafenib treatment both in vitro and in vivo, whereas ectopic PI16 expression produced the opposite effect. Mechanistically, PI16 could suppress p38 MAPK/caspase‐dependent apoptosis in this process, and p38 MAPK inhibitor reversed the sorafenib sensitive phenotype caused by PI16 inhibition. Clinically, immunohistochemistry was used to detect PI16 levels in resected patients with HCC prior to sorafenib treatment. We showed that high PI16 levels represented an independent risk factor for disease progression in patients treated with sorafenib. Patients with low PI16 showed significantly better progression free survival and overall survival after sorafenib therapy. In conclusion, PI16 attenuates response to sorafenib treatment in HCC, and may be a helpful prognostic biomarker of sorafenib treatment.

## Graphical abstract

PI16 is upregulated in HCC tissues; PI16 knockdown sensitizes HCC cells to sorafenib treatment in vitro and in vivo; PI16 may serve as a predictive marker for sorafenib‐treated patients after resection.

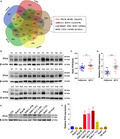

## 1 INTRODUCTION

Liver cancer is reported as the sixth most commonly diagnosed malignancy and the fourth leading cause of cancer‐related deaths worldwide in 2018, with approximately 841 000 new diagnoses and 782 000 deaths every year.(1) Hepatocellular carcinoma (HCC) comprises more than 75% of all liver cancers.(1) Partial hepatectomy and liver transplantation remain the most effective treatment for patients with HCC. However, due to the late symptom presentation and aggressive tumor biological behavior, many patients with HCC are diagnosed at very advanced stages, when surgical treatments may not be applicable.(2) In these advanced cases, sorafenib becomes the only recommended therapy since it was approved by FDA as the only first‐line drug in 2008. Two randomized clinical trials with large sample size have reported significantly improved overall survival in the sorafenib treatment arm in patients with advanced HCC.(3, 4) However, the survival benefits were modest; the median survival was improved less than 3 months as compared with placebo arm.(3, 4) In the SHARP trial, only 2% patients showed a partial response and not a patient showed a complete response.(4) It is considered that drug resistance might be a main problem leading to unsatisfactory partial response of sorafenib therapy. Low response rates of sorafenib remarkably limits its clinical efficacy.

Sorafenib targets various tyrosine kinases, including vascular endothelial growth factor receptor (VEGFR), platelet‐derived growth factor receptor (PDGFR), and Raf family kinases, to block tumor cell proliferation, and angiogenesis.(5) Since various signaling pathways involved, different mechanisms resulting in sorafenib resistance were found, including compensatory activation of alternative survival pathways,(6, 7) eliciting autophagy to alleviate ER stress‐related apoptosis,(8) and enrichment of the liver cancer stem cells.(9) The precise molecular mechanisms of sorafenib resistance are largely uncovered.(10) In addition, there is no ideally marker to predict the clinical response of sorafenib treatment because it targets multiple kinases. Unlike some other kinase inhibitors, such as tarceva (EGFR inhibitor) or crizotinib (Anaplastic Lymphoma kinase (ALK) inhibitor), their clinical efficacy can be accurately predicted by EGFR or ALK mutations.(11, 12) Therefore, it is urgent to investigate the molecular basis and identify useful biomarkers of sorafenib resistance, which can predict the outcome and improve the clinical benefits of sorafenib treatment.

In this study, we performed comprehensive analysis of TCGA datasets of five different adenocarcinomas and identified PI16 as one of the top differential genes. Peptidase inhibitor 16 (PI16) is localized to chromosome 6p21.2 in human,(13) which is also called as prostate secretory protein 94‐binding protein (PSPBP). It is a member of the cysteine‐rich secretory proteins, antigen 5, and pathogenesis‐related1 proteins (CAP) superfamily.(13) The function of PI16 appears to be complicated and largely not understood, and there are only a few researches studying PI16. Reeves et al(14) reported that PI16 is a helpful prognostic marker post radical prostatectomy in patients with prostate cancer. However, the expression and function of PI16 in HCC are not investigated so far as we know.

In summary, we investigated the exact function of PI16 and found it was associated with sorafenib‐induced apoptosis in HCC. PI16 inhibition could improve the sensitivity to sorafenib treatment by suppressing p38 MAPK/caspase‐dependent apoptosis in vitro and in vivo. Importantly, analyses of the clinical information suggested that PI16 might be a predictive biomarker for the efficacy to sorafenib treatment.

## 2 MATERIALS AND METHODS

### 2.1 Patients

All HCC samples and paired normal tissues were obtained at Shanghai General Hospital from 2010 to 2016. Samples used to perform PI16 immunochemistry staining were retrieved from resected patients who went on receiving sorafenib treatment at initial dose of 400 mg. All sorafenib‐treated patients underwent dynamic computed tomographic scanning before treatment, at 1 month after initiating the sorafenib treatment, and every 3 months thereafter. Patients’ response to sorafenib was assessed according to the modified response evaluation criteria in solid tumors (mRECIST).(15) Informed consent was obtained from each patient. This study was approved by ethics committee of Shanghai General Hospital under the guidelines of the Declaration of Helsinki.(16)

### 2.2 Quantitative real‐time PCR

Extraction of total RNA from snap frozen liver tissue was performed with TRIzol (Invitrogen, Carlsbad, CA, USA). Synthesis of cDNA was conducted using Superscript III reverse‐transcription reagent (Invitrogen) with 1 μg RNA. Quantitative real‐time PCR was performed with the SYBR Green I dye (Roche, Basel, Switzerland). The PCR cycling started at 95°C for 30 seconds followed by 40 cycles of 95°C for 5 seconds, 60°C for 30 seconds, with a last step at 72°C for 20 minutes. The primer sequences used were as follows: PI16 (forward), 5′‐ATATGGATCCACCATGCACGGCTCC‐3′; PI16 (reverse), 5′‐CGAATTCTCAGAAGATTCCAGCCAACACC‐3′; β‐actin (forward), 5′‐GTGGGGCGCCCCAGGCACCA‐3′; β‐actin (reverse), 5′‐CTCCTTAAGTCACGCACGATTTC‐3′. Relative mRNA levels changes were analyzed using 2^−ΔΔCt^ method.

### 2.3 Western blotting

Liver specimens or cell cultures were lysed in RIPA buffer (Thermo Fisher scientific, Waltham, MA, USA), mixed with proteases inhibitors (Roche), for 30 min at 4°C, and then, centrifuged at 13 000 rpm at 4°C for 10 min. Protein samples were separated using SDS‐PAGE and then, transferred onto PVDF membranes. After blocking, the membranes were incubated with primary antibodies (anti‐PI16 antibody, anti‐β‐actin antibody, anti‐Cleaved caspase‐3 antibody: Abcam, Cambridge, UK; anti‐PARP antibody, anti‐p38 antibody, anti‐p‐p38 antibody, anti‐AKT antibody, anti‐p‐AKT antibody: Cell Signaling Technology, Danvers, MA, USA) at 4°C overnight. Then the membranes were washed with TBST (20 mM Tris‐HCl, 150 mM NaCl, 1% Tween‐20) and incubated with HRP‐conjugated secondary antibodies (1:5000; Abcam, Cambridge, MA, USA) for 1 hour. The protein signals were revealed using enhanced chemiluminescence (Merck Millipore, Billerica, MA, USA). All western blots were conducted at least three times, and the images are representative of consistent results.

### 2.4 Cell culture and transfection

HEK293T cells, MHCC‐97H cells, and HepG2 cells were cultured using Dulbecco's Modified Eagle's Medium (DMEM; Gibco BRL, Grand Island, NY, USA) containing 10% fetal bovine serum (FBS; Gibco BRL) and 1% penicillin/streptomycin (HyClone, South Logan, UT, USA). All cells were maintained at 37°C in a 5% CO_2_ incubator. HEK293T, MHCC‐97H cells and HepG2 cells were transfected with the plasmids using polythylenimine (Polyscience, Warrington, PA, USA) and lipofectamine^TM^ 2000 (Invitrogen) reagents according to the manufacturers' protocol. For stable cell lines, the lentivirus technology was conducted for overexpression and knockdown of PI16 gene. In brief, lentivirus plasmids were co‐transfected using the packing plasmids ∆8.9 and VSVG into HEK293T cells with a ratio of 10:9:1. The viral supernatants were collected 2 days post transfection and used to infect MHCC‐97H cells and HepG2 cells along with polybrene (Sigma‐Aldrich, St. Louis, MO, USA), and then, subjected to selection with puromycin until uninfected cells were eliminated.

### 2.5 Immunohistochemistry

Formalin‐fixed paraffin‐embedded HCC tissues and mice tissues were prepared. After deparaffinize and rehydrate, 5 µm thick slides were stained with hematoxylin & eosin (H&E) or primary antibodies (anti‐PI16 antibody: Abcam, Cambridge, UK; anti‐Cleaved caspase‐3 antibody, anti‐Ki67 antibody: Cell Signaling Technology, Danvers, MA, USA), followed by incubation with horseradish peroxidase (HRP)‐conjugated secondary antibody (Abcam, Cambridge, MA) and then, DAB substrate kit (Abcam, Cambridge, MA). The cells stained brown were considered as positive.

### 2.6 Immunofluorescence staining

TUNEL staining was conducted with TUNEL assay kit (Roche) following antigen retrieval and permeabilization. Nucleus labeled with DAPI (Sigma‐Aldrich) appears blue, and TUNEL‐positive apoptosis cells labeled with FITC are green. All the slides were visualized using immunofluorescence microscopy (Olympus, Tokyo, Japan).

### 2.7 Apoptosis assay

Apoptosis was measured with Annexin V‐PI Apoptosis Detection Kit (BD Biosciences, San Jose, CA, USA) accordingly. Cells were seeded in six‐well plates and treated with different concentrations of sorafenib. After 2 days incubation, cells were washed using phosphate‐buffer solution (PBS) and resuspended in annexin‐binding buffer, followed by Annexin V and PI reagents staining for 15 minutes in the dark. Flow cytometry was performed to test apoptosis in these cells. All flow cytometry data were analyzed using FlowJo software (Tree Star, Ashland, OR, USA).

### 2.8 LDH leakage assay

Cytotoxicity induced by sorafenib was also evaluated by lactate dehydrogenase (LDH) assay. The culture medium was centrifuged to get a cell free supernatant. LDH activity in the culture medium was analyzed with a commercially available kit (Sigma‐Aldrich) according to the manufacturer's protocol.

### 2.9 Scratch wound‐healing assays

Cells were cultured and grown to nearly 90% confluence on six‐well plates. A scratch was generated down the center of well using a sterile pipette tip, and then, washed with PBS once. Images of the wound closure were obtained at different time points and the widths were quantified as compared with baseline values.

### 2.10 CCK‐8 assays

CCK‐8 (Dojindo, Kumamoto, Japan) assays were performed to evaluate cell proliferation. Cells were seeded in 96‐well plates at a density of 2000 cells/well. Absorbance at 450 nm was determined with a spectrophotometer at different time points.

### 2.11 Colony formation assay

Under the initial density of 1000 cells per well, cells were seeded into six‐well plates. Colonies were identified by 0.1% crystal violet (Sigma‐Aldrich) staining after approximately 14 days in culture.

### 2.12 Transwell migration assay

Cells were seeded in the upper chamber (6 × 10^4^ cells) in 200 μL FBS‐free medium. A total of 800 μL of DMEM (Gibco BRL) containing 10% FBS (Gibco BRL) was added into the lower chamber. After the cells were incubated for 24 hours, cells adhered to the low surface of the plates were fixed, stained, and counted in randomly selected fields.

### 2.13 Animal studies

Five‐ to six‐week‐old male BALB/C nude mice were obtained from Shanghai SLAC Laboratory (Shanghai, China). Approximately 1 × 10^6^ cells (PI16 knockdown MHCC‐97H cells or its negative control, n = 5/each group) resuspended in 100 μL PBS were injected subcutaneously into either flank of the mice. Intragastric administration of sorafenib (30 mg/kg) was performed every day since the formation of palpable tumors. The tumor size was tested every day and its volume was calculated following the formula: length × width^2^ × 0.5. At 6 weeks post‐inoculation, the tumors were surgically harvested for histological analysis. The protocol was approved by Institutional Animal Care and Use Committee of Shanghai General Hospital.

### 2.14 Statistical analysis

Continuous data were shown as the mean ± SD while discrete variables were shown as frequencies. Categorical variables were compared using Pearson's chi‐squared test or Fisher's exact test, and continuous variables were calculated using two‐tailed Student's t test. Variables with statistical significance were analyzed by the forward stepwise multivariate logistic regression analysis. Survival rates were assessed using Kaplan‐Meier analysis and differences between subgroups were compared using the log‐rank test. All statistical analyses were performed with GraphPad Prism 5 (GraphPad Software, La Jolla, CA, USA). Statistical significance was established as *P* < .05. The significance is shown as follows: *P < .05, **P < .01, *** *P* < .001.

## 3 RESULTS

### 3.1 PI16 is over expressed in HCC

**Figure 1:**
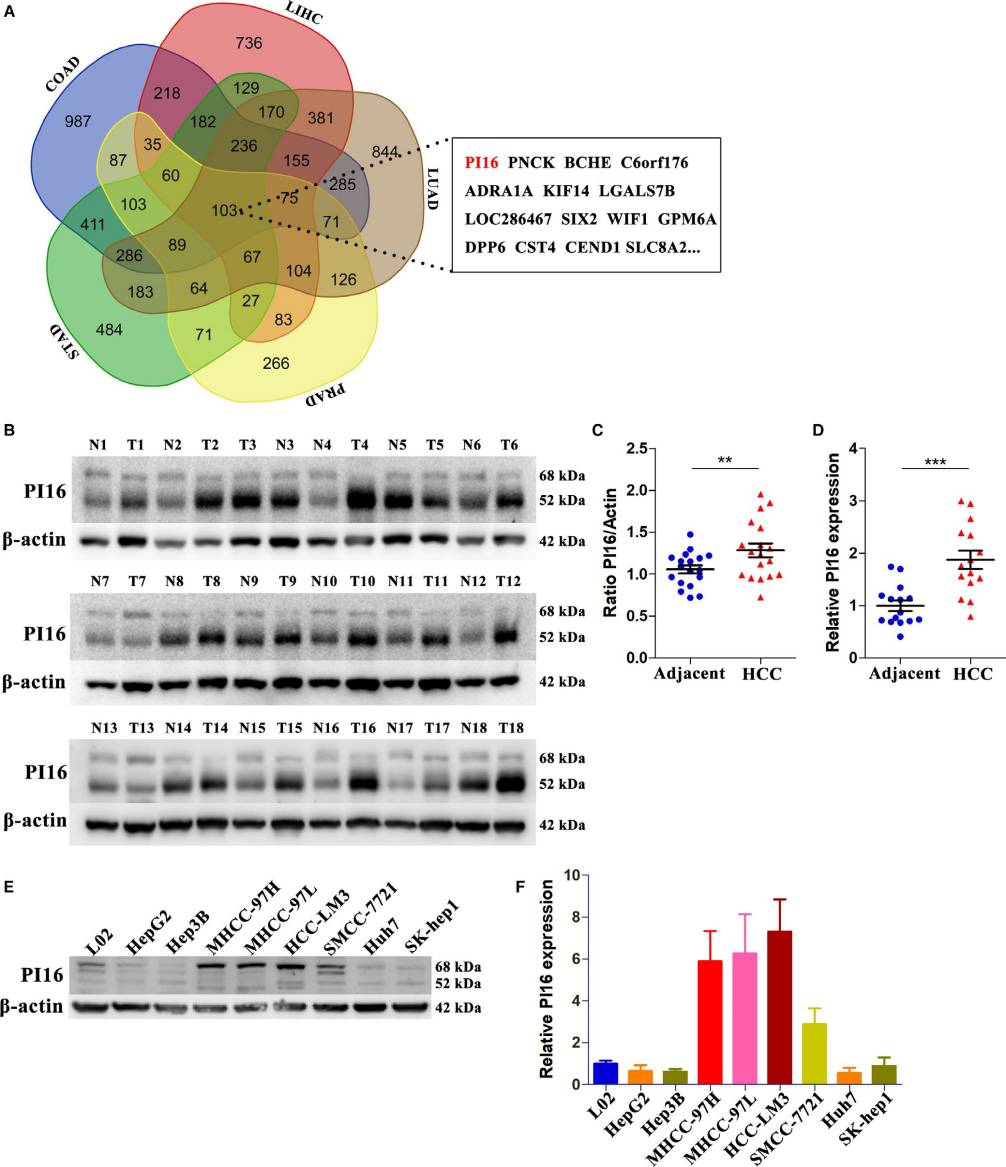
PI16 expression is increased in HCC tissues. (A) Venn diagram indicating the identification of differentially expressed genes (fold change > 2.0, Student's t test, *P* < .05) in five different adenocarcinomas. (B and C) The protein levels of PI16 in 18 HCC tissues and paired normal tissues determined by Western blot; β‐actin was used as a control. (D) The mRNA levels of PI16 in 15 HCC tissues and paired normal tissues determined by qRT‐PCR. (E) The protein levels of PI16 in normal hepatocytes and HCC cell lines determined by Western blot; β‐actin was used as a control. (F) The mRNA levels of PI16 in normal hepatocytes and HCC cell lines determined by qRT‐PCR. **P < .01, ***P < .001, Student's t test

Comprehensive analysis of TCGA datasets of five different adenocarcinomas including liver hepatocellular carcinoma (LIHC), colon adenocarcinoma (COAD), lung adenocarcinoma (LUAD), stomach adenocarcinoma (STAD), and prostate adenocarcinoma (PRAD), identified 103 differentially expressed genes (fold change > 2.0, *P* < .05) (Figure 1A). The list of used TCGA datasets were shown in the heatmap of each adenocarcinoma (Figure S1). The list of all differential genes obtained was presented in Table S1. The results indicated PI16 as one of the top differential genes, then we validated the bioinformatics data in a sample cohort consisting of 18 pairs of HCC tissues by Western blots and qRT‐PCR. The protein level of PI16 was significantly over expressed in HCC tissues vs adjacent normal tissues (Figure 1B and C). The mRNA level of PI16 was tested in 15 pairs of tissues since three of them were not qualified after quality control. The results of qRT‐PCR also showed increased PI16 mRNA level in HCC tissues (Figure 1D). Since PI16 is also a secretory protein,(14) we analyzed serum PI16 levels of patients with these adenocarcinomas and healthy controls. However, some patients with these adenocarcinomas tended to have higher levels of PI16 in the serum than healthy controls, but it did not reach statistical significance (Figure S2). Finally, we investigated the levels of PI16 protein (Figure 1E) and PI16 mRNA (Figure 1F) in human normal hepatocytes (L02) and various HCC cell lines. PI16 levels were upregulated in some HCC cell lines including MHCC‐97H, MHCC‐97L, HCC‐LM3, and SMCC‐7721, as compared with L02 cells.

### 3.2 PI16 inhibition sensitize HCC cells to sorafenib treatment

**Figure 2:**
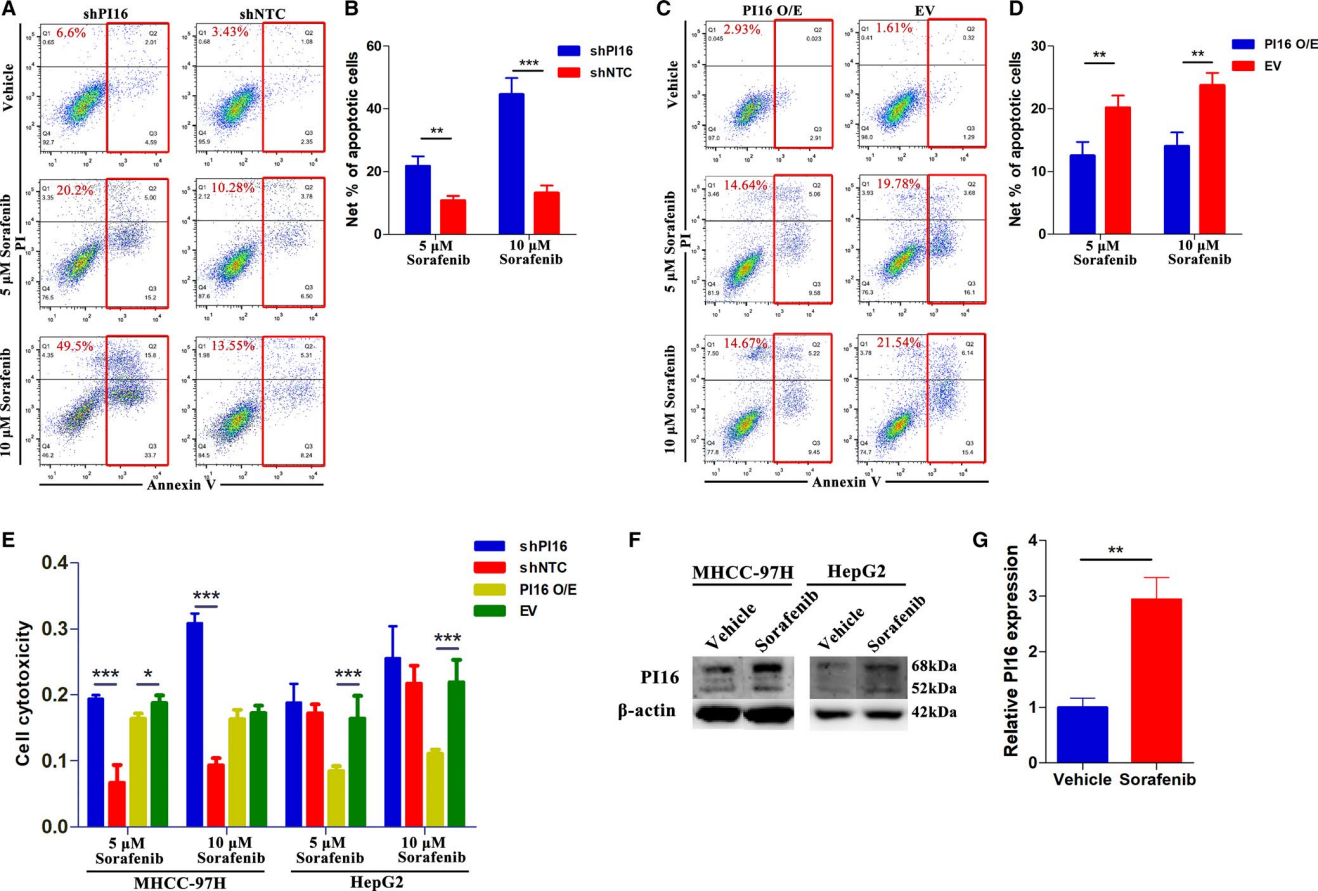
PI16 knockdown potentiates sorafenib response in HCC. (A and B) Representative flow cytometry images of Annexin V‐PI staining and quantification of net apoptosis in PI16 knockdown MHCC‐97H cells treated with 5 μM Sorafenib and 10 μM Sorafenib. (C and D) Representative flow cytometry images of Annexin V‐PI staining and quantification of net apoptosis in PI16 overexpressed HepG2 cells treated with 5 μM Sorafenib and 10 μM Sorafenib. (E) Cell cytotoxicity LDH assay of PI16 knockdown, overexpression and negative controls in both 97H and HepG2 cells treated with 5 μM Sorafenib and 10 μM Sorafenib. (F and G) PI16 expression in wild‐type MHCC‐97H and HepG2 cells after 5 μM Sorafenib treatment; β‐actin was used as a control. **P < .01, ***P < .001, Student's t test

To clarify the role of PI16 in HCC, we investigated its potential function. According to PI16 protein levels of HCC cell lines (Figure 1E), wo stably transfected MHCC‐97H and HepG2 cells with a PI16‐specific shRNA and established stable PI16 overexpressed MHCC‐97H and HepG2 cells. The knockdown and overexpression level of PI16 in MHCC‐97H and HepG2 cells were detected using western blot (Figure S3A). Additionally, the green fluorescent protein was utilized to tag the transfection, which showed a high transfer efficiency (Figure S3D). In concentrations of both 5 μM sorafenib and 10 μM sorafenib, we observed that HCC cells in which PI16 was suppressed were more sensitive to sorafenib treatment in MHCC‐97H cells (Figure 2A and B); while PI16 overexpression in HepG2 cells could increase resistance (Figure 2C and D). In PI16 overexpressed MHCC‐97H cells (Figure S3B) and PI16 knockdown HepG2 cells (Figure S3C), no significant differences were observed. In line with this, cell cytotoxicity LDH assay showed that PI16 knockdown sensitized MHCC‐97H cells to sorafenib treatment, and PI16 overexpression in HepG2 cells increased its resistance (Figure 2E). Additionally, we also observed increased protein (Figure 2F) and mRNA (Figure 2G) levels of PI16 after sorafenib treatment in both wild‐type MHCC‐97H and HepG2 cells. However, wound‐healing assays, CCK8 assays, transwell assays, and colony formation assays showed no significant differences between PI16 knockdown or overexpression cell model and relative controls (Figure S3). Thus, these results confirmed a link between PI16 and sorafenib response in HCC.

### 3.3 PI16 inhibition promotes sorafenib‐induced apoptosis via p38 MAPK/caspase activation

**Figure 3:**
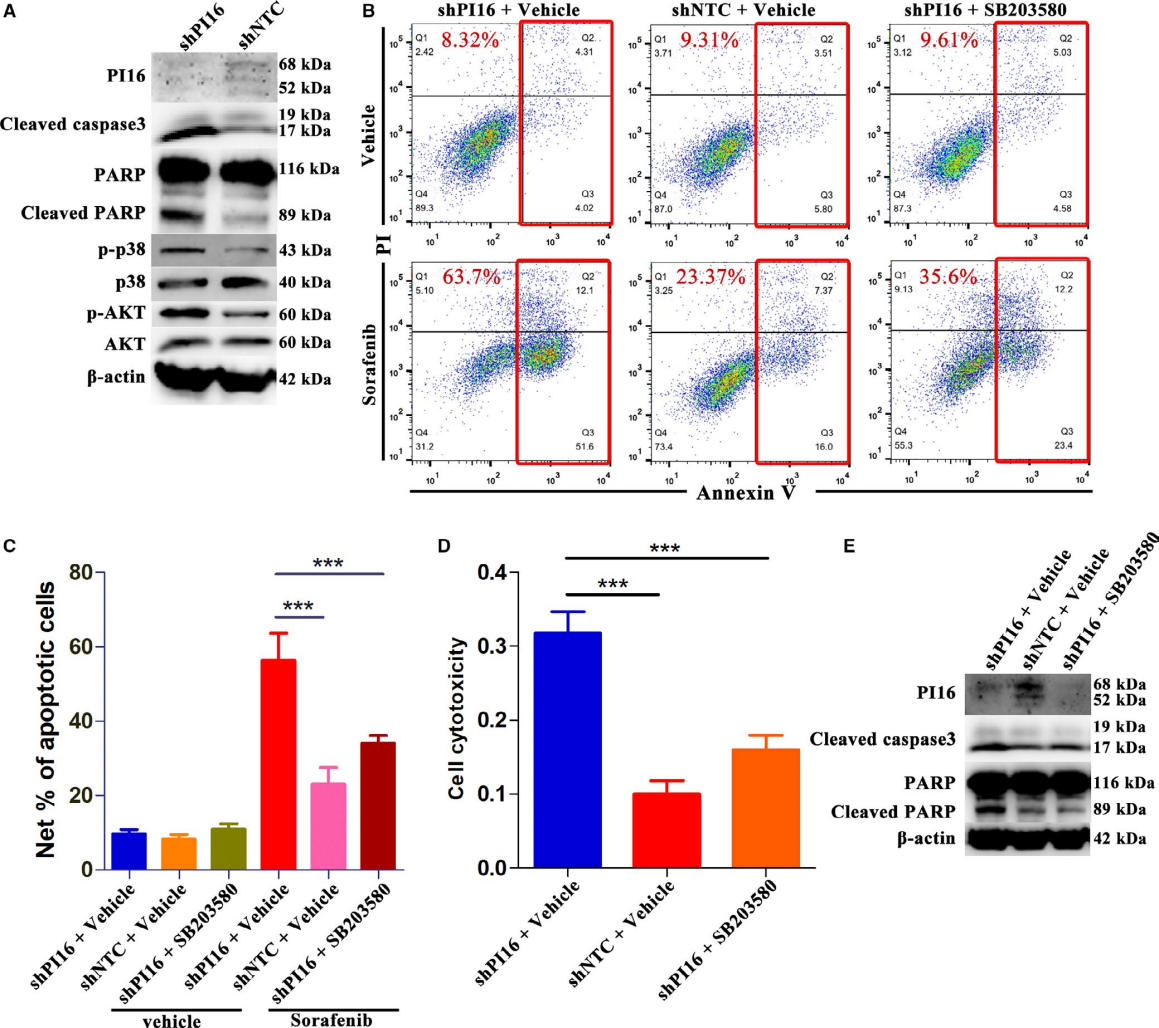
PI16 knockdown improves sorafenib response in HCC via activating p38 MAPK/caspase‐dependent apoptosis. (A) Representative markers of apoptosis, PI3K/AKT and MAPK signaling in PI16 knockdown MHCC‐97H cells treated by 5 μM Sorafenib determined by Western blot; Total AKT, total p‐p38 MAPK, and β‐actin were used as controls. (B and C) Representative flow cytometry images of Annexin V‐PI staining and quantification of net apoptosis in PI16 knockdown MHCC‐97H cells treated with 5 μM Sorafenib, and in the absence or presence of SB203580. (D) Cell cytotoxicity LDH assay of PI16 knockdown MHCC‐97H cells treated with 5 μM Sorafenib, and in the absence or presence of SB203580. (E) Protein levels of cleaved caspase 3 and cleaved PARP determined by Western blot in PI16 knockdown MHCC‐97H cells treated with 5 μM Sorafenib, and in the absence or presence of SB203580; β‐actin was used as a control. ***P < .001, Student's t test

Pathway analysis (gene set enrichment analysis) using the TCGA LIHC datasets mentioned before indicated significant associations among activation of apoptosis, MAPK, and PI3K/AKT signaling and PI16 in HCC (Figure S4). Accordingly, to study the underlying mechanisms, representative markers of these pathways were examined. As shown in Figure 3A, western blot analysis revealed that PI16 knockdown generated higher expressions of Cleaved caspase 3 and Cleaved PARP in MHCC‐97H cells treated with sorafenib. Besides, PI16 knockdown MHCC‐97H cells also showed increased levels of p‐p38 MAPK and p‐AKT after sorafenib treatment.

To determine whether p38 MAPK is a major downstream mediator of PI16, SB203580, an inhibitor of p‐p38 MAPK, was utilized. Similar apoptosis and LDH cell toxicity assay were performed in the absence of or presence of SB203580. In terms of phenotypes, addition of SB202190 in PI16 knockdown MHCC‐97H cells, significantly led to them regaining the resistance to sorafenib treatment (Figure 3B‐D). Consistently, inhibition of p‐p38 MAPK in PI16 knockdown MHCC‐97H cells also decreased the levels of Cleaved caspase 3 and Cleaved PARP. Consequently, these results indicated that PI16 inhibition attenuated sorafenib resistance by activating p38 MAPK/caspase signaling.

### 3.4 PI16 inhibition is effective in suppressing tumor growth in vivo

**Figure 4:**
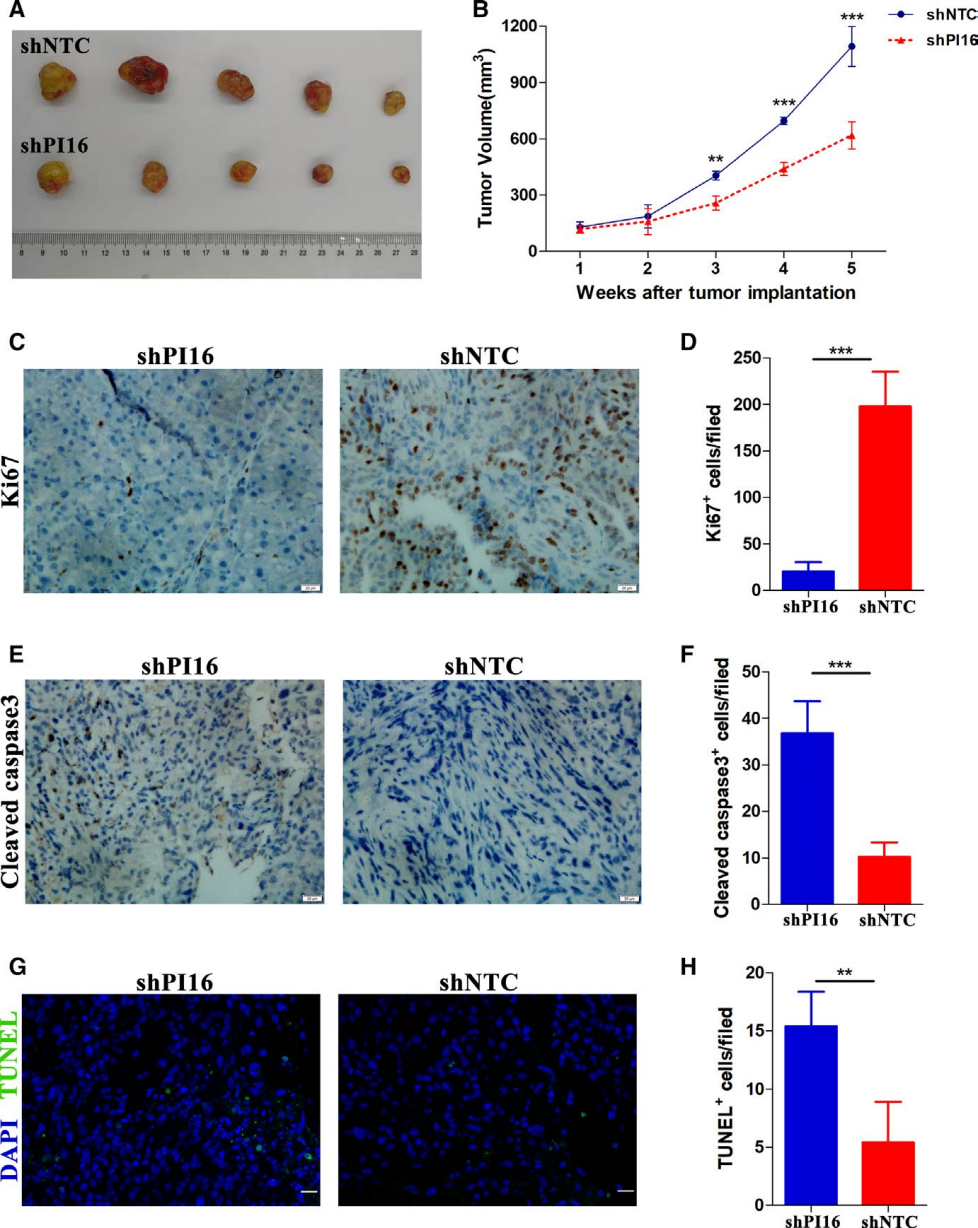
PI16 inhibition increases the sensitivity of HCC xenografts to sorafenib. (A and B) MHCC‐97H cells stably transfected with the PI16 shRNA or empty vector were inoculated subcutaneously in nude mice, and then, treated by sorafenib as described in methods. (C and D) Representative immunohistochemical staining images of the proliferation marker Ki‐67 and quantitative analysis. Scale bar = 20μm. (E and F) Representative immunohistochemical staining images of the apoptosis marker Cleaved caspase‐3 and quantitative analysis. Scale bar = 20μm. (G and H) Representative TUNEL immunofluorescence staining images and quantitative analysis. Scale bar = 20μm. **P < .01, ***P < .001, Student's t test

To further confirm the role of PI16 on sorafenib resistance, we conducted subcutaneous tumor xenograft assays using PI16 knockdown MHCC‐97H cells and its negative control cells in BALB/c nude mice, which were subsequently treated with sorafenib. Representative images of nude mice were shown in Figure S5A. The mouse weight baseline characteristic between the two groups was comparable (Figure S5B). The tumors generated by PI16 knockdown MHCC‐97H cells showed significant smaller volumes than that by the controls after sorafenib treatment (Figure S4A). The tumor growth rate and size without sorafenib treatment between the two groups were comparable (Figure S5C). From the 3rd week after sorafenib treatment, the shPI16 group showed obvious decrease in tumor size as compared with the controls (Figure 4B). Subsequent immunohistochemical staining showed that xenograft tumors with PI16 knockdown had significant lower levels of Ki67 than negative controls did after sorafenib treatment (Figure 4C and D). In terms of apoptosis, tumors with PI16 knockdown showed significant more Cleaved caspase‐3 positive (Figure 4E and F) and TUNEL positive (Figure 4G and H) cells per field than controls after sorafenib treatment.

### 3.5 PI16 represents a predictive biomarker in sorafenib‐treated patients with HCC

Given the effects of PI16 on sorafenib resistance in HCC cells and mice, we further investigated the clinical significance of PI16 in patients’ response to sorafenib. HCC tissues were obtained from resected patients who received sorafenib treatment thereafter. Patients were grouped by sorafenib response. Disease control group included patients with stable disease (SD), partial response (PR), and complete response (CR), while disease progression group include patients with progressive disease (PD). Patients characteristics were analyzed in Table 1.

**Table 1:** Characteristics of the patients with HCC according to Sorafenib response

	Disease control (n = 26)	Disease progression (n = 37)	Univariate, *P* value	Multivariate, *P* value; OR (95% CI)
Age, year	51.5 ± 7.5	49.6 ± 8.3	0.37	
Gender (male/female)	22/4	33/4	0.71	
Underlying liver disease, no (%)				
HBV	22 (84.6%)	31 (83,8%)		
HCV	1 (3.8%)	2 (5.4%)		
Chronic alcoholism	3 (11.5%)	3 (8.1%)		
Autoimmune hepatitis	0 (%)	1 (2.7%)	0.92	
Child‐Pugh score, (A/B)	23/3	31/6	0.73	
BCLC staging, (B/C)	12/14	5/32	0.008**	0.022*; 3.92 [1.31‐12.86]
AFP > 400 ng/ml, no (%)	12 (46.2%)	21 (56.8%)	0.45	
Diameter of largest tumor > 3 cm, no (%)	17 (65.4%)	30 (81.1%)	0.24	
Tumor number > 5, no (%)	13 (50%)	26 (70.3%)	0.12	
PVTT, no (%)	1 (3.8%)	8 (21.6)	0.07	
Extrahepatic metastasis, no (%)	6 (22.2%)	19 (51.4%)	0.036*	0.573; 1.47 [0.41‐5.73]
High PI16 expression, no (%)	10 (38.5%)	26 (70.3%)	0.019**	0.037*; 2.83 [1.16‐11.49]

Note

AFP, alphafetoprotein; HBV, hepatitis B virus; HCV, hepatitis C virus; HCC, hepatocellular carcinoma; OR: odds ratio; CI: confidence interval.

The significance of bold values are shown as follows: *P < .05, **P < .01.

**Figure 5:**
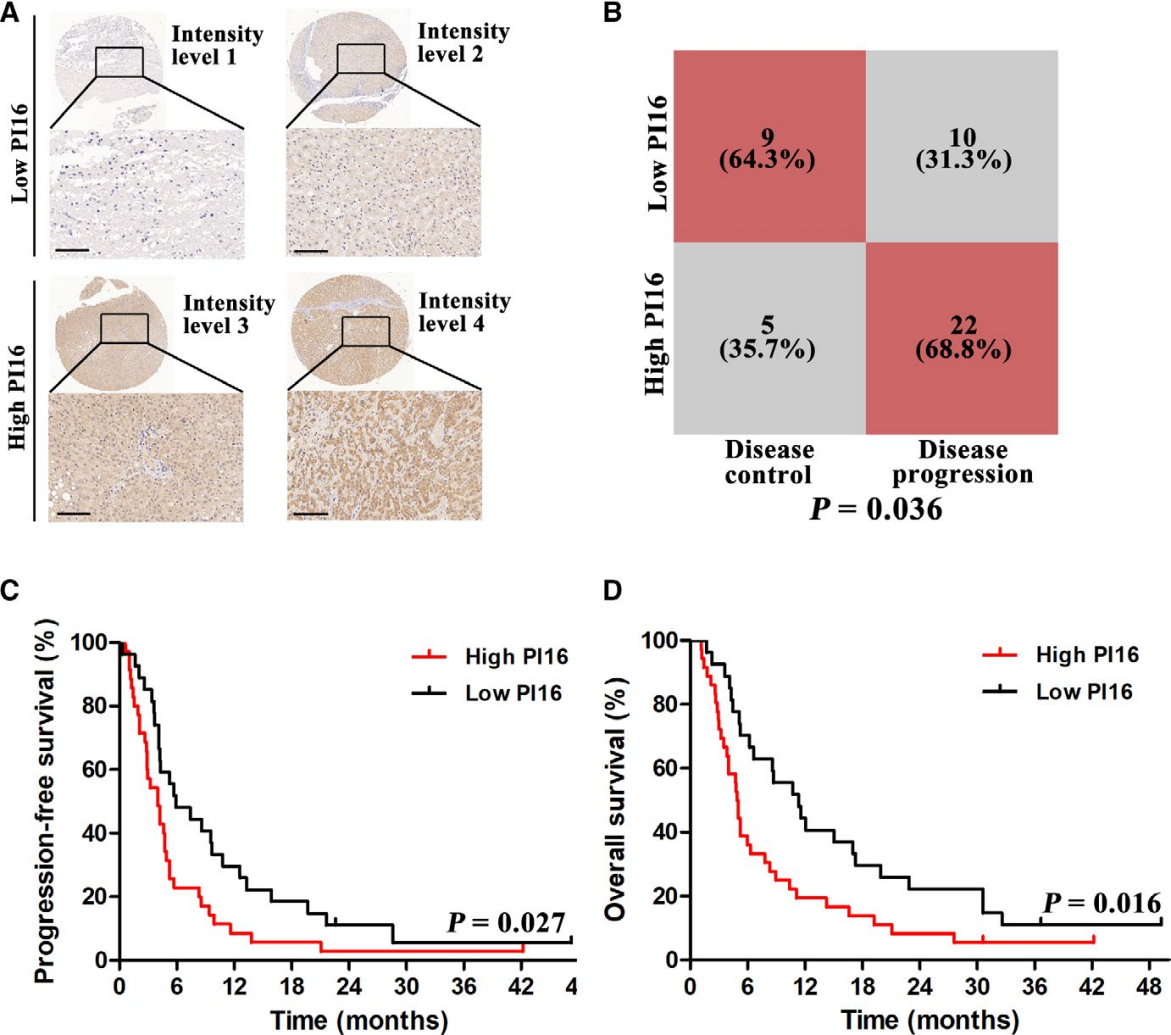
PI16 may be a predictive marker for sorafenib treated patients after resection. (A) Representative images of PI16 immunohistochemical staining at different intensity levels. Scale bar = 100μm. (B) Association between PI16 and Sorafenib response in BCLC staging C patients. (C) Kaplan‐Meier analysis of PFS grouped by high PI16 and low PI16 levels. (D) Kaplan‐Meier analysis of OS grouped by high PI16 and low PI16 levels

Different intensity levels of PI16 staining were showed in Figure 5A; level 1 and level 2 were considered as low PI16, while level 3 and level 4 were high PI16. As shown in Table 1, there were more patients of BCLC staging C, more patients with extrahepatic metastasis, and more patients with high PI16 in disease progression group than in disease control group. Next, we performed multivariate analysis using BCLC staging, extrahepatic metastasis and PI16 level as covariables. BCLC staging C (OR = 3.92; 95% CI: 1.31‐12.86, *P* = .022) and high PI16 (OR = 2.83; 95% CI: 1.16‐11.49, *P* = .037) were revealed as independent risk factors for disease progression, respectively.

Since sorafenib is generally recommended in BCLC staging C patients, we further performed subgroup analysis in these patients. As shown in Figure 5B, for BCLC staging C patients, there were a significantly higher proportion of high PI16 in patients with disease progression (68.8%) than those with disease control (35.7%); For BCLC staging B patients, the proportions were 80% vs 41.7%, however which did not reach statistical significance (Figure S6).

In survival analysis, patients with low PI16, compared with patients with high levels, had significantly longer PFS (median, 5.7 vs 3.6 months, *P* = .027; Figure 5C) and OS (median, 10.3 vs 4.9 months, *P* = .016; Figure 5D). Altogether, these results suggested that PI16 could predict sorafenib treatment outcome.

## 4 DISCUSSION

Sorafenib was the only first‐line therapy approved by FDA for patients with advanced HCC from 2008 to 2018. Until now, it remains the most common used standard of care for these patients. However, the clinical benefit was very limited due to poor response and modest increased survival.(4) Of note, the treatment response varies remarkably among patients.(17) Therefore, it is necessary to elucidate the molecular mechanisms of sorafenib resistance and search for helpful biomarkers of sorafenib sensitivity.

In this study, we identified the differential gene, PI16, using TCGA datasets of five different adenocarcinomas. Next, we validated that PI16 expression was increased in HCC compared to adjacent normal tissues. PI16 was firstly described as a serum protein released by prostate cells with high binding affinity for PSP94, which was originally used to recognize the free and total forms of PSP94.(14, 18) It then became clear that PI16 had a wide expression profile. PI16 is expressed in kidney, stomach, liver, colon, small intestine, and many other organs,(13) and in various cell types, including immune cells,(19) and cardiac cells.(20) However, the exact function of PI16 was not well studied. Reeves et al(14) reported that PI16 was an independent prognostic marker after radical prostatectomy in patients with prostate cancer. As far as we know, there are no studies investigating PI16 in other cancers, and underlying mechanisms remain obscure. We also tested the serum levels of PI16 in patients with different adenocarcinomas including prostate cancer. Unlike the differences between solid tumors and paired normal tissues, it showed no statistical significance in serum PI16 levels between patients with these adenocarcinomas and healthy controls (Figure S2). This might be caused by a small sample size and different background of patients.

The next gain‐ and loss‐of‐function assays confirmed that PI16 could potentiate sorafenib resistance in HCC cells, and PI16 inhibition effectively suppressed tumor growth in mice. We showed that PI16 knockdown significantly improved sorafenib sensitivity in HCC cells at two different concentrations. Besides, PI16 overexpression or knockdown in HCC cells did not significantly affect proliferation, migration or invasion (Figure S3). Mechanistic studies found PI16 to suppress p38/caspase‐dependent apoptosis. Various studies reported that p38 MAPK activation augments apoptosis in response to antitumor drugs in many cancers. Cheng X et al reported that p38 MAPK activation played an important role in their complexes‐mediated apoptosis in pancreatic cancer cells.(21) Pereira et al reported that p38 MAPK inhibition sensitized tumor cells to cisplatin‐induced apoptosis in breast and colon cancer cells.(22) It is also reported that cyclophosphamide could induce apoptosis through activating p38 MAPK pathway.(23) In HCC, some studies demonstrated the pro‐apoptotic role of p38 MAPK. Bao et al(24) reported that Huaier polysaccharide‐induced apoptosis in HCC cells through activating p38 MAPK. Liu et al(25) showed that aspafilioside B could activate p38 MAPK, which consequently induced apoptosis in HCC cells. Chiu et al(26) reported that p38 MAPK could promote apoptosis in Naphtho[1,2‐b] furan‐4,5‐dione‐treated HCC cells. In addition to in vitro studies, some in vivo studies also reported the pro‐apoptotic role of p38 MAPK. Iyoda et al(27) showed that reduction of the p38 MAPK could result in the resistance to apoptosis in human HCC. More recently, Tong et al(28) found that inhibition of p38 MAPK activation could suppress sorafenib‐induced apoptosis in HCC. Out findings were consistent with these studies. However, in another in vitro study, inhibition of p38 MAPK activation increased tumor necrosis factor‐induced apoptosis.(29) In our opinion, this difference might be caused by these different agents used. The exact role of p38 MAPK on apoptosis could be dependent on its regulator and cellular context. In our study, p38 MAPK inhibitor reversed the sorafenib sensitive phenotype caused by PI16 inhibition, which confirmed PI16 as an upstream regulator.

Sorafenib has been used as first‐line therapy in patients with advanced HCC, renal cell carcinoma for more than 10 years and very recently in patients with advanced thyroid cancer.(30) However, after more than 10 years of study of sorafenib, there are still no validated predictive markers or factors of sorafenib sensitivity in HCC.(31) Even though sorafenib targets multiple kinases, such markers should exist considering its remarkable heterogeneous clinical response. Here we showed, patients with low PI16 levels had significantly longer PFS and OS. In multivariate analysis, high PI16 expression was confirmed as an independent risk factor of disease progression. According to our study, PI16 could represent as a biomarker to stratify patients with HCC to sorafenib therapy, which might improve clinical efficacy of sorafenib and deserved to be tested in futured prospected trials.

There were two major limitations of our study. First, although we showed significant apoptotic phenotype of PI16 and the pathway it targets, the exact mechanism remains unclear. PI16, almost an unknown protein in cancer research, needs further deeper investigations. Second, this study was retrospective and single‐center in nature, which needed be validated by high‐quality prospective studies.

In summary, we elucidated the critical role of PI16 in sorafenib response in HCC, wherein it targets p38 MAPK. Furthermore, PI16 represented an independent predictive factor for both sorafenib response and long‐term prognosis. Further clinical evaluation of PI16 should be conducted, which may help stratifying patients to sorafenib treatment and maximize its clinical efficacy.

## CONFLICTS OF INTEREST

The authors declare no potential conflicts of interest.

## AUTHOR CONTRIBUTION

Lin Zhong and Hao Li proposed the study. Pusen Wang, Zhongyi Jiang, and Xueni Liu performed the research and wrote the first draft. Pusen Wang, Kanru Yu, and Chunguang Wang collected and analyzed the data. All authors contributed to the design and interpretation of the study and to further drafts.

## Supporting information

Fig S1

Fig S2

Fig S3

Fig S4

Fig S5

Fig S6

Table S1

## Data Availability

The data that support the findings of this study are available on request from the corresponding author.
