# Supplementary figures and images for: PI16 attenuates response to sorafenib and represents a predictive biomarker in hepatocellular carcinoma
Source: Cancer Med. 2020 Aug 10;9(19):6972–83. doi: 10.1002/cam4.3331 (PMC7541153; doi:10.1002/cam4.3331)

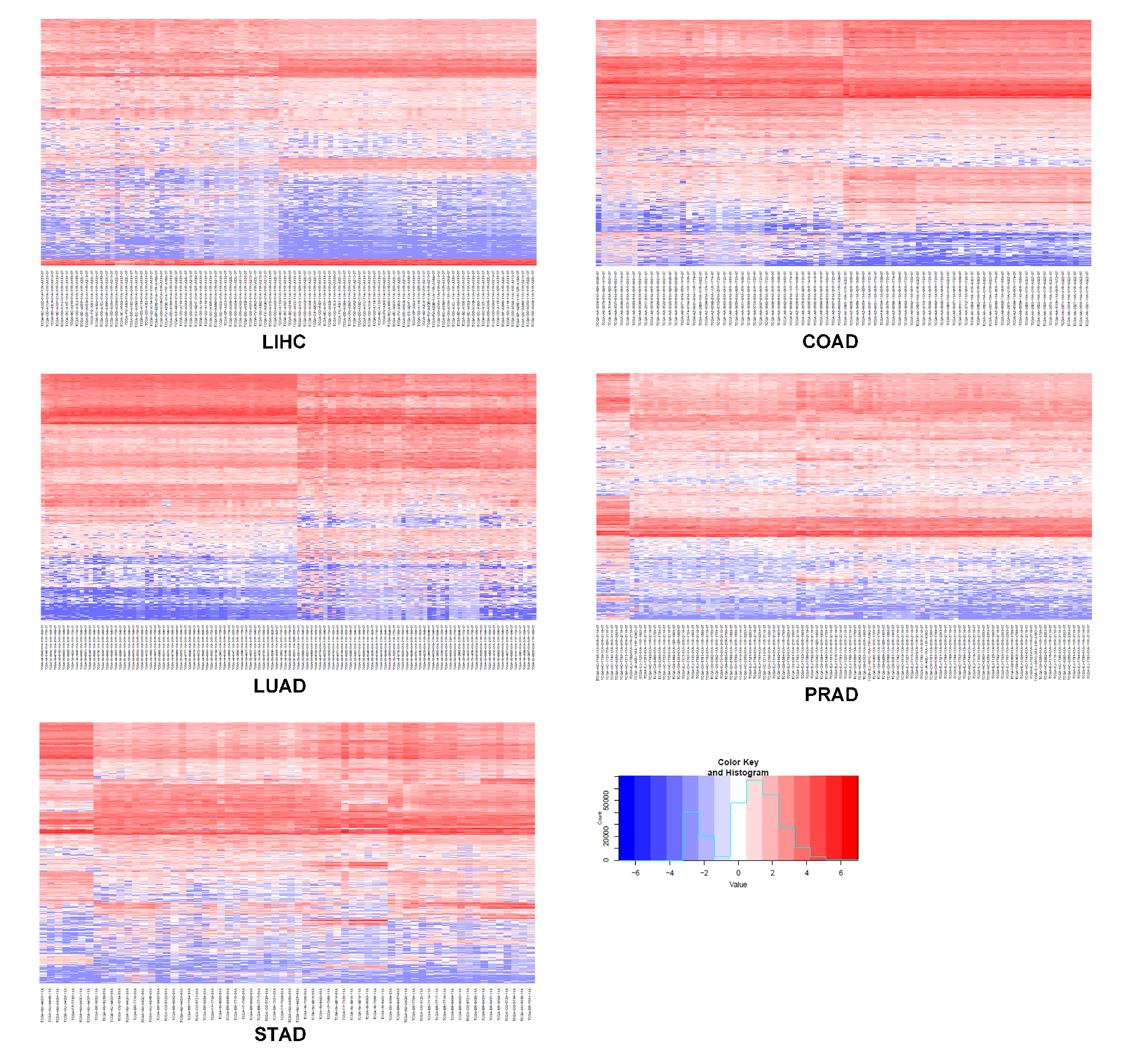

Supplement: Supplementary file 1 — Fig S1 [file CAM4-9-6972-s001.tif]

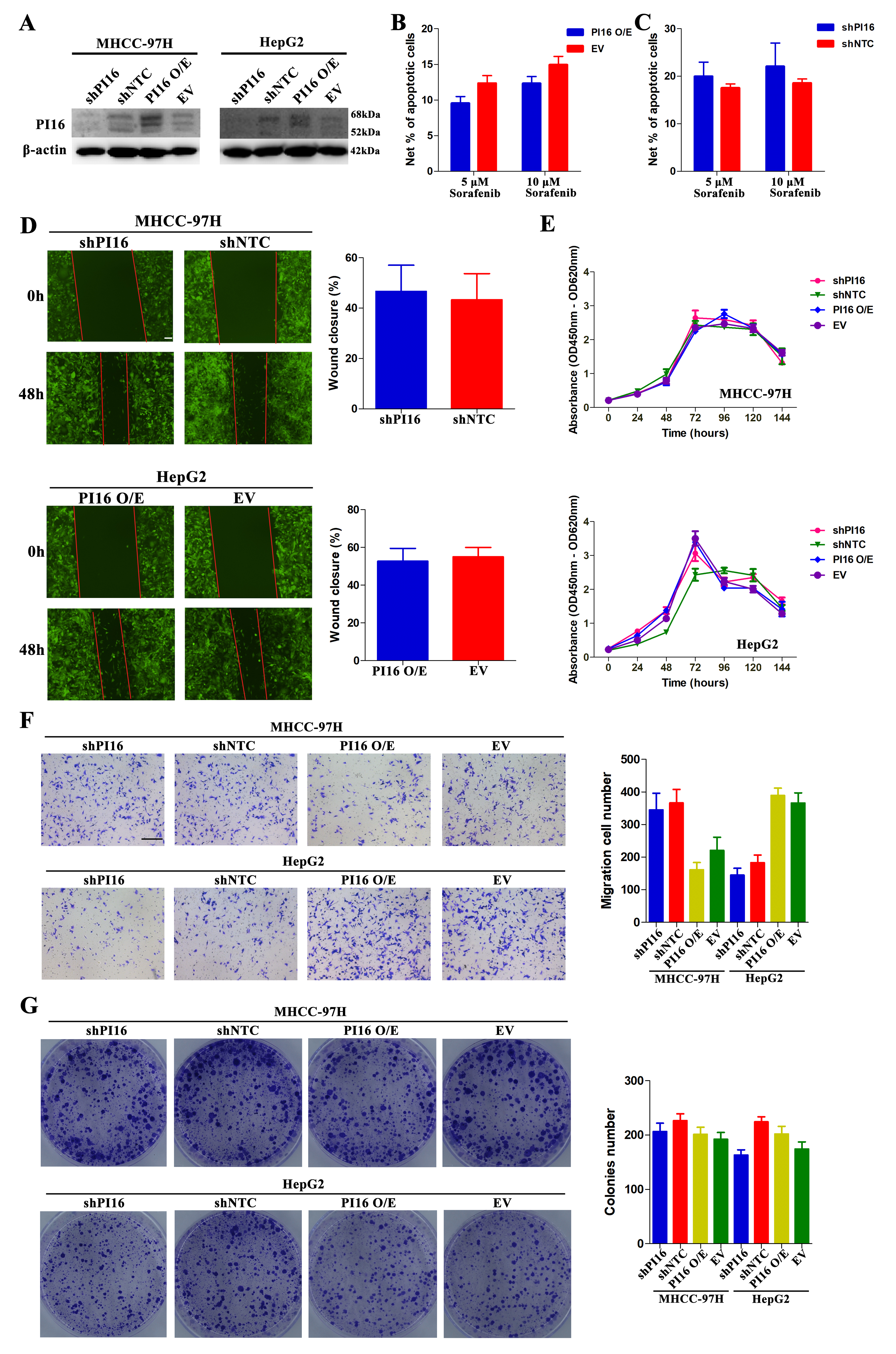

Supplement: Supplementary file 3 — Fig S3 [file CAM4-9-6972-s003.tif]

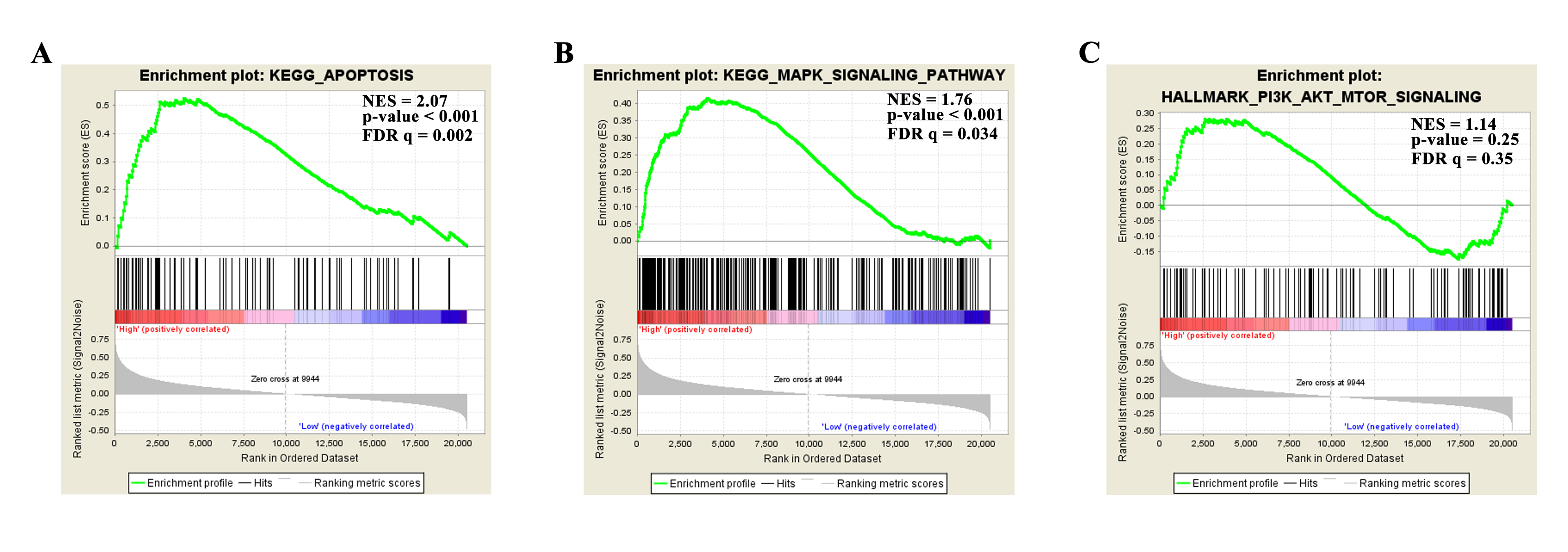

Supplement: Supplementary file 4 — Fig S4 [file CAM4-9-6972-s004.tif]

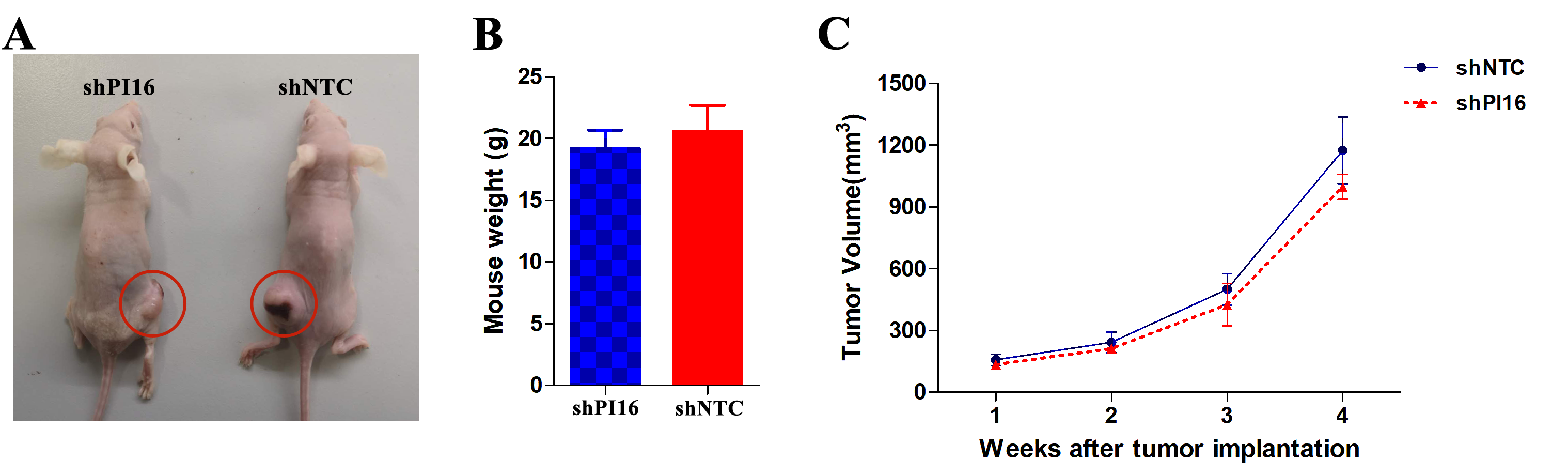

Supplement: Supplementary file 5 — Fig S5 [file CAM4-9-6972-s005.tif]

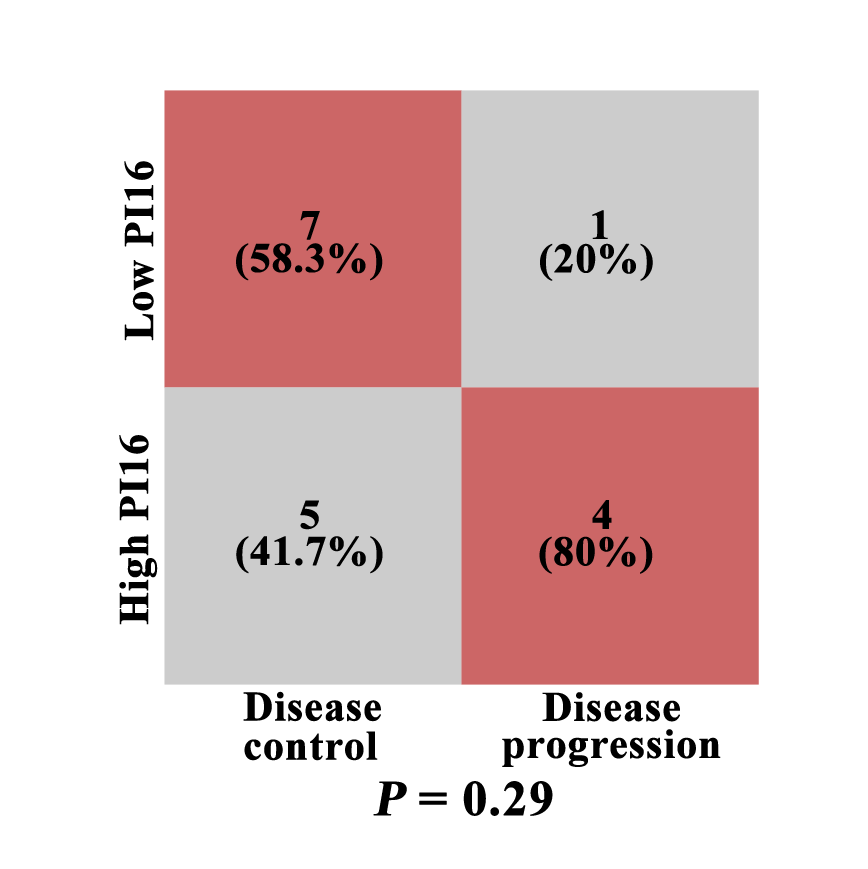

Supplement: Supplementary file 6 — Fig S6 [file CAM4-9-6972-s006.tif]
